# Berberine chloride pretreatment exhibits neuroprotective effect against 6-hydroxydopamine-induced neuronal insult in rat

**Published:** 2015

**Authors:** Feraidoon Negahdar, Mehdi Mehdizadeh, Mohammad Taghi Joghataei, Mehrdad Roghani, Fereshteh Mehraeen, Ehsan Poorghayoomi

**Affiliations:** a *Cellular and Molecular Research Center, Department of Anatomical Sciences, and Faculty of Advanced Technology, Iran University of Medical Sciences, Tehran, Iran. *; b *Neurophysiology Research Center, Shahed University, Tehran, Iran.*

**Keywords:** Berberine, Parkinson’s disease, 6-hydroxydopamine, Tyrosine hydroxylase

## Abstract

Parkinson’s disease (PD) is a rather common movement disorder as a result of the degeneration of dopaminergic neurons within the substantianigra. Current treatments for PD afford symptomatic relief with no prevention of disease progression. Due to the neuroprotective and anti-apoptotic potential of the isoquinoline alkaloid berberine (BBR), this study was conducted to assess whether BBR pretreatment could attenuate behavioral and neuronal derangement in 6-hydroxydopamine (6-OHDA)-induced model of PD in the rat. Unilateral intrastriatal 6-OHDA-lesioned rats received BBR at doses of 25 and/or 50 mg/kg (i.p.) three times at an interval of 24 h, started 2 days before the surgery. After 1 week, apomorphine caused significant contralateral rotations and a significant reduction in the number of Nissl-stained and tyrosine-hydroxylase (TH)-positive neurons on the left side of the substantianigra. BBR pretreatment at a dose of 50 mg/kg significantly reduced rotations and prevented loss of TH-positive neurons. These results indicate pre-lesion administration of BBR could protect against 6-OHDA toxicity and this may be of benefit besides other available therapies in PD.

## Introduction

Parkinson’s disease (PD) is due to degeneration of dopaminergic neurons in the mesencephalicsubstantianigra. Clinical debilitating manifestations of PD include resting tremor, muscle rigidity, postural imbalance, an inability to initiate movements, and a lack of facial expression (1). The neurotoxin 6-hydroxydopamine (6-OHDA) has routinely been used to injure nigral dopaminergic neurons. Intrastriatal injection of 6-OHDA has been used as one of the valuable models of PD (2). Activity of tyrosine hydroxylase (TH), the rate-limiting enzyme in the synthesis of the dopamine, progressively decreases following the loss of dopaminergic neurons in PD (3). Intracerebral injection of 6-OHDA also leads to apoptotic cell death of dopaminergic neurons in the substantianigra, thus, it is implicated in the pathogenesis of Parkinson’s disease (4, 5). Meanwhile, oxidative stress-induced neuronal death plays a pivotal role in pathogenesis of neurodegenerative disorders like PD (6). Currently, treatment of PD is symptomatic and begins with levodopa or dopamine agonists, depending on age and neuropsychiatric symptoms (7). Nevertheless, after a certain time period, most of the patients experience some side effects including motor and non-motor fluctuations and dyskinesia (8).

Natural compounds with antioxidant activity from plants were reported to reduce oxidative stress burden via scavenging reactive oxygen species, to chelate deleterious metal ions like iron and to diminish inflammation and in this way could attenuate nigrostriatal dopaminergic neuronal loss in PD (9). Berberine (BBR) is a benzyl tetra isoquinoline alkaloid and the primary pharmacological active constituent of *Coptidisrhizoma*, which could inhibit neuronal apoptosis in cerebral ischemia (10). Chronic BBR treatment of streptozotocin-diabetic rats could ameliorate learning and memory impairment due to improvement of synaptic dysfunction and anti-apoptotic property (11). BBR also protects against ischemic brain injury by decreasing oxidative stress and inhibiting mitochondrial apoptosis (12). BBR is also a potent suppressor of neuroflammation and could be of therapeutic potential for the treatment of neurological and neurodegenerative diseases like Alzheimer’s disease (13). Post-lesion treatment of 6-hydroxydopamine (6-OHDA)-lesioned rats with BBR did not show any improvement in apomorphine-induced rotations and was not capable of restoring dopamine level and tyrosinehydroxylase level (14). In contrast, BBR has been able to protect human dopaminergic neuronal cells against 6-OHDA neurotoxicity through the induction of heme oxygenase-1 (15). Thus, the present study was undertaken to investigate theneuroprotective effect of pre-lesion BBR treatment in 6-OHDA induced model of PD using behavioral and histochemical methods.

## Experimental

*Animals*

Adult male Wistar rats, weighing 210-260 g (n = 60) (procured from Pasteur’s Institute, Tehran) were housed in a temperature-controlled colony room under light/dark cycle with free access to food and water. The used procedures for animals and their care were according to NIH guidelines and the protocol was approved by Ethics committee of Iran University of Medical Sciences (Tehran, Iran). The animals were held in the colony room for at least one week before being tested. Those rats with net rotations less than 30/hour following intraperitoneal injection of apomorphine hydrochloride (2 mg/kg; Sigma Chemical, USA) according to an earlier study (6) were selected for the present study. The animals were randomly divided into six groups: sham-operated, BBR25 and BBR50-treated sham-operated groups, lesion group (6-OHDA) and BBR25 and BBR50-treated lesion groups (6-OHDA + BBR25 and 6-OHDA + BBR50). Unilateral intrastriatal 6-OHDA (Sigma Chemical, USA) injection (left side) was performed through a 5 µl Hamilton syringe on anesthetized rats (ketamine 80 mg/kg and xylazine8 mg/kg, i.p.) using stereotaxic apparatus (Stoelting, USA) at the coordinates: L–3 mm, AP 9.2 mm, V 4.5 mm from the center of the interaural line, according to the atlas of Paxinos and Watson (16). At the end of injection, the needle was left in place for an additional 5 min and then withdrawn at a rate of 1 mm/min. The lesion group received a single injection of 5 µl of 0.9% saline containing 2.5 µg/µl of 6-hydroxydopamine-HCL (Sigma Chemical, USA) and 0.2% ascorbic acid (W/V) at a rate of 1 µl/min. The sham group received an identical volume of ascorbate-saline solution. The 6-OHDA + BBR25 and 6-OHDA + BBR50 groups received the neurotoxin in addition to BBR IP dissolved in propylene glycol (Merck, Germany) at doses of 25 or 50 mg/kg, respectively. BBR (Sigma Chemical, USA) was daily administered, started two days before the surgery, with the last injection being 1 h before the surgery.

*Behavioral evaluation*

The animals were tested for rotational behavior by apomorphine hydrochloride (2 mg/kg, i.p.) one week before surgery (baseline) and after 1 week. The rotations were measured according to a method as described previously (2). Briefly, the animals were allowed to habituate for 10 min and then 1 min after the injection, full rotations were counted in a cylindrical container (a diameter of 33 cm and a height of 35 cm) at 10-min intervals for 60 min in a dimly-lighted and quiet room. Net number of rotations was defined as the contralateral scores minus the ipsilateralones.

*Histochemical study*

At the end of the study, the rats were deeply anesthetized with a high dose of ketamine (150 mg/kg) and perfused through the ascending aorta with 50-100 ml of 0.9% saline followed by 100-150 ml of fixative solution containing 4% paraformaldehyde in 0.1 M phosphate buffer (PB, pH 7.4) followed by 100 ml of 0.1 M PB. Following perfusion, the brains were removed from the skull, blocks of forebrain and brainstem were prepared, and after final steps of preparation (immersion in 30% sucrose solution for 1-2 days), sections were cut at a thickness of 30 µm on a freezing microtome (Leica, Germany) and collected in PB (0.1 M). Every second section was Nissl-stained with 0.1% cresyl violet (Sigma). The other sections were used for tyrosine hydroxylase immunohistochemistry.

*Tyrosine hydroxylase (TH) immunohistochemistry*

Tyrosine hydroxylase (TH) is the rate limiting enzyme for dopamine synthesis and is usually used as a selective marker of dopaminergic neurons. The sections on gelatin-coated slides were incubated in 0.1% sodium borohydride in phosphate-buffered saline (PBS, pH 7.4), methanol containing 0.03% H2O2, then in Triton X100 (0.03%) and 0.1% bovine serum albumin in PBS, with rinsing 2-3 times between the steps in a humidified chamber at darkness at room temperature. Then, sections were incubated with primary polyclonal rabbit anti-TH antibody (1/200) in PBS at darkness overnight and then with secondary anti-rabbit IgG-peroxidase antibody (HRP bound) raised in goat (1/200) in PBS for two hours. To reveal the bound peroxidase, the sections were incubated in diaminobenzidinetetrahydrochloride (10 mg/20 ml of PBS and 0.03% H2O2) for 5-8 min, rinsed, dehydrated, cleared and coverslipped.

*Histological evaluation*

For each animal, mesencephalic sections (interaural 2.9-4.2 mm) were examined by a method as described previously (6). Briefly, Nissl-stained neurons and TH-positive neurons of the SNC were manually counted (Light microscopy; X400) using a superimposed grid to facilitate the procedure. At least two sections representative of each of four Paxinos-Watson planes (4.2, 3.7, 3.2, 2.97; Interaural) were examined by scanning the entire extent on each side. Counting was done blind to the treatments received.

*Statistical analysis*

All data were expressed as meansstandard error. For statistical evaluation of rotational behavior and histochemical data, the parametric one-way ANOVA test followed by Tukey’s*post-hoc* test was used. In all analyses, the null hypothesis was rejected at a level of 0.05.

## Results and Discussion

**Figure 1:**
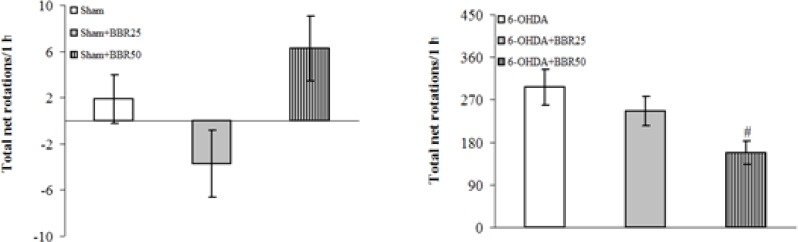
Total net number of apomorphine-induced rotations/1 h after 1 week in sham (upper panel) and 6-OHDA (lower panel) groups

The beneficial effect of BBRat doses of 25 and 50 mg/kg was evaluated on apomorphine-induced rotations for a period of 1 hour (Figure 1). There were no significant differences among the groups at baseline (before surgery). Statistical analysis of the total net number of rotations 1 week after the surgery showed that apomorphine caused a very significant contralateral turning in the rats of 6-OHDA-lesioned group (p < 0.0001) and induced less significant rotations in 6-OHDA + BBR25 and 6-OHDA + BBR50 groups (p < 0.0005 and p < 0.005, respectively) in comparison with Sham group. Moreover, the group 6-OHDA + BBR50 showed a significant reduction of rotations (*p *< 0.05) when compared to 6-OHDA-lesioned group.

The positive values indicate contralateral rotations. 6-OHDA stands for the neurotoxin 6-hydroxydopamine. # p < 0.05 (versus 6-OHDA).

**Figure 2:**
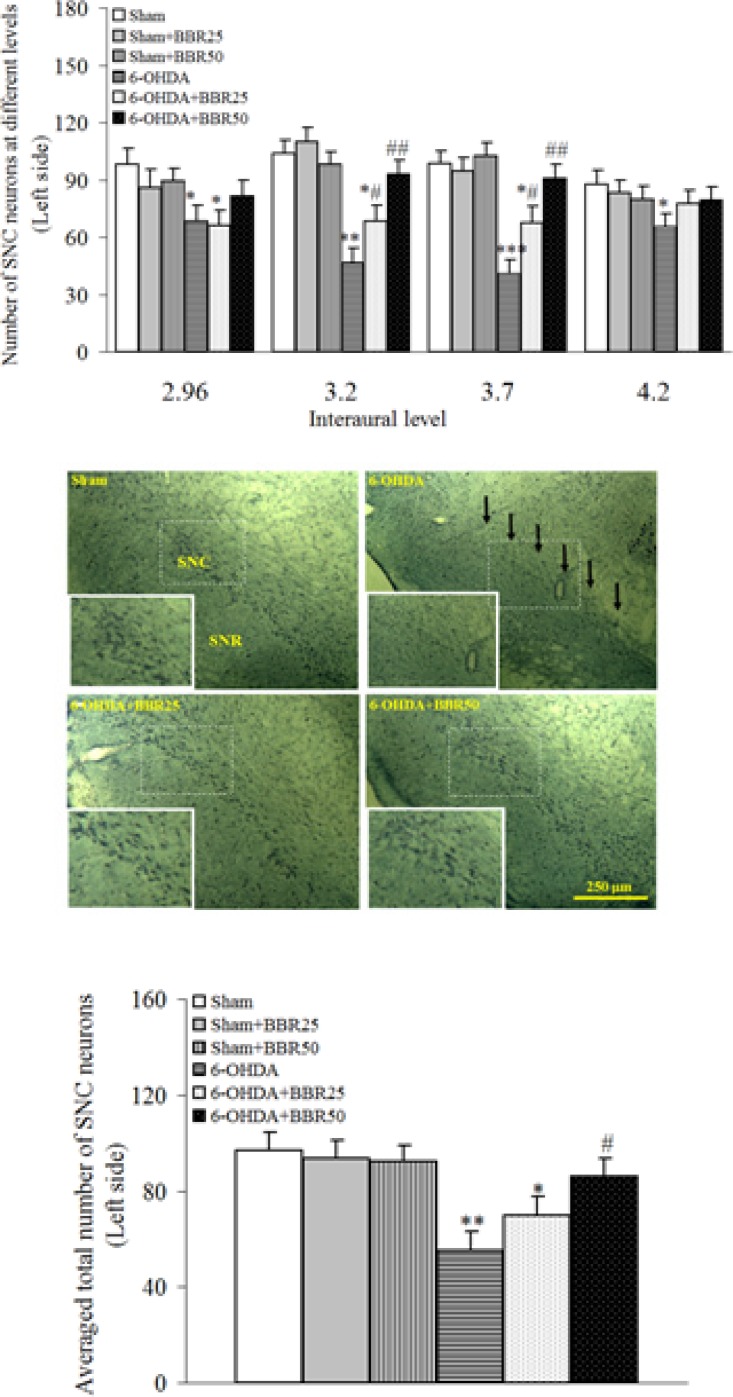
Total number of Nissl-stained neurons on the left side of substantianigra pars compacta (SNC) at different interaural planes (upper panel) and its averaged number at all planes (lower panel) 1 week after the surgery

The results of histochemical studies in Nissl staining (Figure 2) showed that there is no significant difference amongst sham, sham + BBR25, and sham + BBR50 groups regarding the number of Nissl-stained neurons on the left side of SNC. In addition, a significant reduction was noticed in 6-OHDA-lesioned group (p < 0.01) and there wasnosignificant reduction in 6-OHDA + BBR50 when compared to sham group. In this respect, the number of Nissl-stained neurons on the left side of SNC was significantly higher in 6-OHDA + BBR50 versus 6-OHDA-lesioned group (p < 0.05).

Photomicrographs show Nissl-stained neurons in coronal sections through the midbrain in experimental groups. A severe reduction in the number of neurons in SNC was observed in the 6-OHDA-lesioned group, but no such marked reduction was noted in the BBR50-pretreated 6-OHDA group as compared to Sham group. A higher magnification photo was also provided for each photomicrograph as inset. (SNC and SNR = Substantianigra pars compacta and pars reticulate, respectively).^*^p < 0.05,^ **^p < 0.01, *** p <0.005 (versus Sham); #p < 0.05, ## p < 0.01 (versus 6-OHDA).

**Figure 3:**
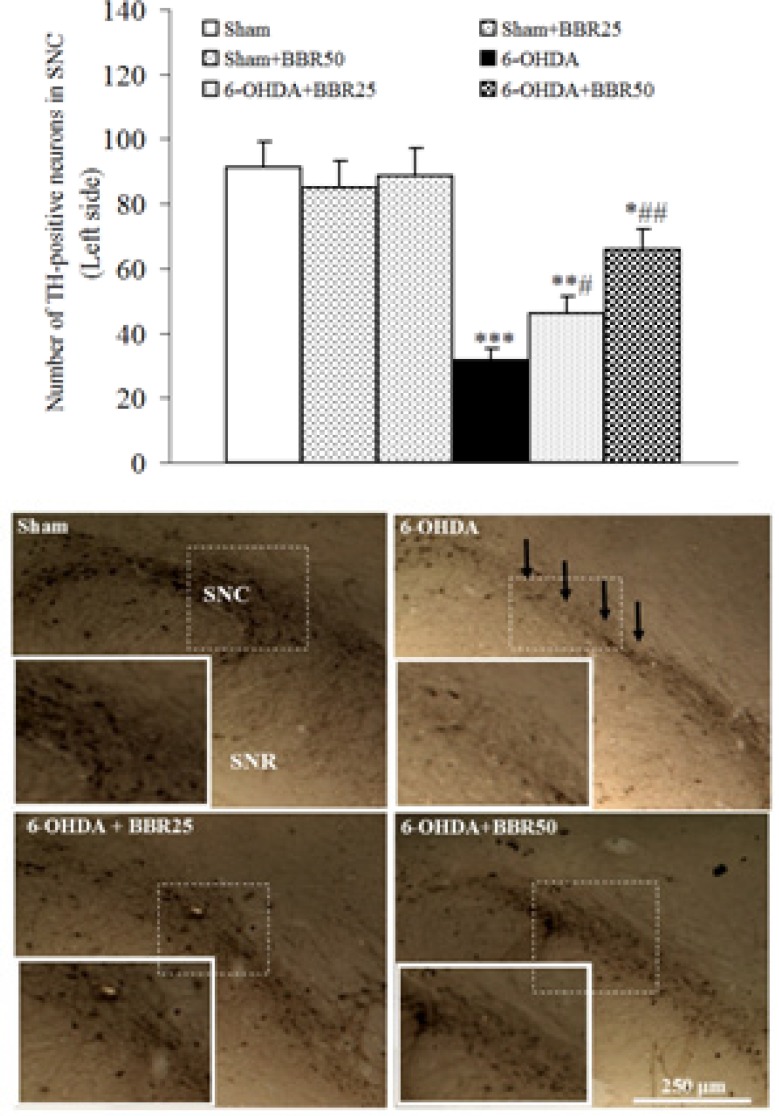
Averaged total number of TH-positive neurons on the left side of substantianigra pars compacta (SNC) 1 week after the surgery (upper panel) and midbrain photomicrographs showing TH-positive immunoreactive neurons within SNC. Arrows show reduction of TH-positive neurons in 6-OHDA-lesioned group. A higher magnification photo was also provided for each photomicrograph as inset

^*^p < 0.05,^ **^p < 0.01, *** p < 0.005(versus Sham); #p < 0.05, ## p < 0.01 (versus 6-OHDA).

The results of TH-immunohistochemical staining (Figure 3) showed that there is no significant difference amongst sham, sham + BBR25, and sham + BBR50 groups regarding the number of TH-positive neurons on the left side of SNC. In addition, a significant reduction was observed in 6-OHDA-lesioned group (p < 0.01) and a less significant reduction in 6-OHDA + BBR25 (p < 0.01) and 6-OHDA + BBR50 when compared to sham group. In this respect, the number of TH-positive neurons on the left side of SNC was significantly higher in 6-OHDA + BBR50in comparison with 6-OHDA-lesioned group (p < 0.01).

In this study, we demonstrated that BBRpretreatment at a dose of 50 mg/kg could significantly decrease apomorphine-induced rotations, attenuate loss of Nissl-stained SNC neurons and prevent the reduction of TH immunoreactivity in SNC dopaminergic neurons.

The preferentialdamage of dopaminergic neurons in patients with PD is attributed to a genetically and/or environmentally-induced neurodegenerative process (17). In addition, 6-OHDA, which is generally used for PD induction in rats, is assumed to cause selective degeneration of dopaminergic neurons (18). The unilateral damage of the nigrostriatal dopaminergic system through intrastriatal injection of 6-OHDA is followed by a reduction in the striatal dopamine level and an upregulation of dopaminergic postsynaptic receptors at the same side. These changes produce a prominent functional and motor asymmetry that can be evaluated by direct-acting dopaminergic agonists like apomorphine (19). These rotations are considered as reliable indicators of nigrostriatal dopamine depletion (20). In this research study, a significant attenuation of the apomorphine-induced rotational behavior was observed in BBR50-pretreated 6-OHDA-lesioned group after 1 week. The observed attenuation of rotational behavior in BBR50-pretreated 6-OHDA group could be due to theneuroprotective effect of BBR against SNC neurodegeneration and maintenance of striatal dopamine at a level that is not accompanied with a marked rotational behavior. In other words, nigrostriatal neurons within SNC were mainly preserved in the presence of BBR against neurodegenerative effects induced by the neurotoxin 6-OHDA.In support of our findings, in an earlier report, BBR was able to protect human dopaminergic neurons against 6-OHDA neurotoxicity through the induction of heme oxygenase-1 (15).

In addition, overproduction of free radicals, especially reactive oxygen speciesisalsoinvolved in 6-OHDA-induced neurodegeneration (20). Oxidative stress is an important factor that could affect the survival of dopaminergic neurons in PD. Neurons mostly depend on energy produced by mitochondria and are simultaneously faced with high levels of reactive oxygen species as well as increased levels of free iron, which can promote hydroxylproduction (21). Overload of the free radical formation may lead to cell death. In addition, auto-oxidation of dopamine may produce dopamine quinine (22). Formation of species such as semiquinones and other free radicals could especially damage nucleic acids, proteins, and membrane lipid components (23). Therefore, the therapeutic approaches are aimed at attenuation of oxidative stress. Free radical scavengers may also be helpful in prolonging survival time of dopaminergic neurons (24). Although in this study, we did not measure oxidative stress markers, but it is possible that BBR could have attenuated neuronal damage and loss through counteracting oxidative stress, possibly via regulating antioxidant defense system as well as inhibition of free radical generation, as shown before (12). This requires further investigation.

Inflammatory process initiated in the brain tissue is also an important causative factor for the PD pathogenesis (25, 26). Pro-inflammatory cytokines released from glial cells could stimulate nitric oxide production and exert a deleterious effect on dopaminergic neurons by activating receptors that contain intra-cytoplasmic death domains involved in apoptotic pathway (27). It has been shown that BBR has anti-inflammatory activity and inhibits lipopolysaccharide-induced inflammatory processes (28). It is possible that BBR may have lowered the level of these inflammatory mediators within the brain, which itself contributes to neuroprotection in 6-OHDA-induced PD model in rats, as observed in our study. Apoptosis is another factor that plays a critical role when cells are exposed to neurotoxins including 6-OHDA (29). BBR could have suppressed 6-OHDA-induced reactive oxygen speciesgeneration and apoptosis (11).

In contrast to our results, a study by Kwon etalhasshown that post-lesion treatment of 6-OHDA-lesioned rats with BBR did not show any improvement in apomorphine-induced rotations and was not capable of restoring dopamine level and tyrosinehydroxylase level (14). In that study, the restorative and not the neuroprotective potential of BBR was evaluated and for this reason, BBR was not capable to improve the condition when used after lesioning the nigrostriatal system. In our study, BBR was administered as a neuroprotective agent before lesioning this system and for this reason, our obtained results are different from those of Kwon *et al*.

In summary, these results indicate that pre-lesion administration of BBR at a dose of 50 mg/kg could protect against 6-OHDA toxicity and this may be of benefit besides other available therapies in PD.
